# Prevalence and Characteristics of Hepatitis B Virus (HBV) Coinfection among HIV-Positive Women in South Africa and Botswana

**DOI:** 10.1371/journal.pone.0134037

**Published:** 2015-07-28

**Authors:** Philippa C. Matthews, Apostolos Beloukas, Amna Malik, Jonathan M. Carlson, Pieter Jooste, Anthony Ogwu, Roger Shapiro, Lynn Riddell, Fabian Chen, Graz Luzzi, Manjeetha Jaggernath, Gerald Jesuthasan, Katie Jeffery, Thumbi Ndung’u, Philip J. R. Goulder, Anna Maria Geretti, Paul Klenerman

**Affiliations:** 1 Nuffield Department of Medicine, University of Oxford, Peter Medawar Building for Pathogen Research, Oxford, United Kingdom; 2 Department of Infectious Diseases and Microbiology, Oxford University Hospitals NHS Trust, John Radcliffe Hospital, Headington, Oxford, United Kingdom; 3 Department of Clinical Infection, Microbiology and Immunology, Institute of Infection and Global Health, University of Liverpool, Liverpool, United Kingdom; 4 Department of Paediatrics, University of Oxford, Peter Medawar Building for Pathogen Research, Oxford, United Kingdom; 5 Microsoft Research, eScience Group, Redmond, Washington, United States of America; 6 Paediatric Department, Kimberley Hospital, Kimberley, Northern Cape, South Africa; 7 Botswana Harvard AIDS Institute Partnership, Botswana; 8 Department of Immunology and Infectious Diseases, Harvard School of Public Health, Boston, Massachusetts, United States of America; 9 Integrated Sexual Health Services, Northampton General Hospital, Cliftonville, Northampton, United Kingdom; 10 Department of Sexual Health, Royal Berkshire Hospital, Reading, United Kingdom; 11 Department of Sexual Health, High Wycombe Hospital, High Wycombe, Buckinghamshire, United Kingdom; 12 HIV Pathogenesis Programme, Doris Duke Medical Research Institute, Nelson R. Mandela School of Medicine, University of KwaZulu-Natal, Durban, South Africa; 13 KwaZulu-Natal Research Institute for Tuberculosis and HIV (K-RITH), University of KwaZulu-Natal, Durban, South Africa; 14 Max Planck Institute for Infection Biology, Chariteplatz, Berlin, Germany; 15 The Ragon Institute of MGH, MIT and Harvard University, Cambridge, Massachusetts, United States of America; 16 NIHR Biomedical Research Centre, John Radcliffe Hospital, Headley Way, Headington, Oxford, United Kingdom; Centers for Disease Control and Prevention, UNITED STATES

## Abstract

There is progressive concern about the evolving burden of morbidity and mortality caused by coinfection with HIV-1 and hepatitis B virus (HBV) in sub-Saharan Africa, but the epidemiology and impact of this problem are not well defined. We therefore set out to assimilate more information about the nature of HBV/HIV coinfection in this region by undertaking a retrospective observational study of southern African adult women. We used samples from previously recruited HIV-1 positive women attending antenatal clinics in three settings in South Africa and Botswana (n = 950) and added a small cohort of HIV-negative antenatal South African women for comparison (n = 72). We tested for HBsAg and followed up HBsAg-positive samples by testing for HBeAg, HBV DNA, HBV genotype, presence of drug-resistance associated mutations (RAMs) and HDV. We identified HBsAg in 72 individuals (7% of the whole cohort), of whom 27% were HBeAg-positive, and the majority HBV genotypes A1 and A2. We did not detect any HDV coinfection. HBV prevalence was significantly different between geographically distinct cohorts, but did not differ according to HIV status. Among adults from South Africa, HBV/HIV coinfected patients had lower CD4+ T cell counts compared to those with HIV-monoinfection (p = 0.02), but this finding was not replicated in the cohort from Botswana. Overall, these data provide a snapshot of the coinfection problem at the heart of the HIV/HBV co-epidemic, and are important to inform public health policy, resource allocation, education, surveillance and clinical care.

## Introduction

There has been a recent revival of political and clinical interest in the problem of infection with Hepatitis B Virus (HBV) in sub-Saharan African populations in whom Human Immunodeficiency Virus (HIV) is also frequently endemic [1, 2]. The progressive availability and success of antiretroviral therapy (ART) has reduced opportunistic infection and malignancy in individuals with HIV, increasing survival and allowing the emergence of previously unrecognized chronic liver disease [3, 4].

Furthermore, there is increasing evidence that chronic HIV/HBV coinfection is associated with long-term morbidity and mortality that exceeds the impact of infection with either one of these viruses alone in African populations [5–8]. This includes evidence of lower CD4+ T cell counts in HIV/HBV coinfected individuals compared to HIV monoinfected patients [8–10]. Adding to the scale of the problem, many individuals in Africa are particularly vulnerable to liver disease for a variety of other reasons including diet, genetics, and exposure to toxins and other pathogens [6, 7, 11].

Despite these concerns, the burden and impact of HIV/HBV coinfection in sub-Saharan Africa have not been well characterized. As reviewed recently [11], there are regional differences in the prevalence and severity of HIV, HBV and hepatitis delta virus (HDV) infection, and the existing literature is generally based on small studies of disparate populations. Although important information can be gained from studying HBV virological markers, such as hepatitis B e-antigen (HBeAg) status, this information is not available for the majority of studies published from African cohorts [11].

We therefore set out to characterize the prevalence and characteristics of HBV infection in a large cohort of HIV-positive African women, and to determine the influence of HBV coinfection on CD4+ T-cell counts and HIV-specific CD8+ T-cell responses using ELISpot assays. We drew upon a large bank of samples that have been gathered over the past decade for studies of HIV infection, as well as adding a small control group of HIV-negative individuals.

We elected to focus on this geographic region as it represents populations at the epicentre of the global HIV epidemic, in whom HBV also represents a substantial and emerging clinical challenge. Our data provide a detailed picture of HBV/HIV in populations who are highly vulnerable to coinfection, for whom resources for screening, diagnosis and treatment are limited.

## Materials and Methods

### Study approach

Over the past decade, our group has produced numerous detailed studies of immune responses to HIV in southern African adults (for examples, see references [12–17]). However, to date, the prevalence and nature of coinfection with other chronic viruses in these cohorts has not been described. When we recently undertook a review of the existing literature surrounding HIV/HBV coinfection in these populations [11], we were alerted to the absence of clear data regarding the prevalence and virologic characteristics of HBV in the region. We therefore set out to characterise the epidemiology and nature of HIV/HBV coinfection in this setting, drawing upon our large pre-existing repository of clinical samples and laboratory data as a starting point for further study.

### Study populations and ethics statement

We performed a retrospective cross-sectional analysis of 1,022 African women, recruited from antenatal and paediatric clinics (in the latter case, as the mothers of HIV-infected children) between 2004 and 2013. Of these, 950 (93%) were HIV-positive, ART-naïve, recruited from four cohorts described as a list below and also in Table 1, with reference to previous descriptions in the published literature where applicable:

- Durban HIV-positive cohort (n = 426): HIV-positive women attending antenatal clinics at Cato Manor and Sinikithemba in Durban, South Africa [18, 19]. Ethics approval was given by the University of KwaZulu-Natal Biomedical Research Ethics Committee (ref. E028/99).
- Kimberley HIV-positive cohort (n = 81): HIV-positive women recruited as mothers of children attending paediatric HIV clinics in Kimberley, South Africa [13, 20]. Ethics approval was given by Ethics Committee of the Faculty of Health Science, University of Free State, Bloemfontein, South Africa (ref. ETOVS Nr 08/09).
- Gaborone HIV-positive cohort (n = 443): HIV-positive women recruited via the Mma Bana Study [21], attending antenatal clinics in Gaborone, Botswana. Ethics approval was given by the Health Research and Development Division, Ministry of Health, Gaborone (ref. PPME-13/18/1).

**Table 1:** Prevalence and characteristics of HBV infection in 1,022 adult women from Botswana and South Africa.

Cohort Name / Location		Masibambisane / Durban	Sinikithemba and Cato Manor / Durban	Kimberley	Mma Bana / Gaborone	Total
**Country of origin**		South Africa	South Africa	South Africa	Botswana	South Africa + Botswana
**Recruitment source**		Antenatal clinics	Antenatal clinics	Paediatric clinics (mothers of HIV-infected children)	Antenatal clinics	All cohorts combined
**HIV-status**		Negative	Positive	Positive	Positive	Mixed
**Number of individuals**		72	426	81	443	1,022
**HIV viral load (RNA copies/ml plasma)**	**Median**	n/a	30,800	33,947	16,400	23,300
	**IQR**	n/a	6,990–112,500	6,400–165,000	3,610–70,100	4,861–93,625
**CD4 T cell count (cells/mm** ^**3**^ **)**	**Median**	n/a	368	325	344	359
	**IQR**	n/a	258–532	225–478	228–1,342	253–507
**Number with HBsAg (% of all individuals)** ^**a**^		6 (8.3)	40 (9.4)	9 (10.8)	17 (3.8)	72 (7.0)
**Number with HBeAg (% of HBsAg+ individuals tested)** ^**a**^		1/6 (16.7)	12/40 (30.0)	1/4 (25.0)	2/10 (20.0)	16/60 (26.7)
**Number with HDV**		0/6 (0)	0/37 (0)	0/8 (0)	0/9 (0)	0/60 (0)

In addition, we investigated a parallel group of HIV-negative women, in order to be able to compare the characteristics of HBV in HIV-positive vs. HIV-negative groups. This group consisted of HIV-negative antenatal women from the Masibambisane cohort, recruited at Prince Mshiyeni Hospital, Durban, South Africa (n = 72). Ethics approval was given by the University of KwaZulu-Natal Biomedical Research Ethics Committee (ref. BF 168.09).

All subjects gave written informed consent for participation.

### HIV-1 RNA load and CD4 T cell count

We quantified HIV-1 RNA from plasma using the Roche Amplicor Version 1.5 assay (Rotkreuz, Switzerland), and measured CD4+ T cell counts by flow cytometry. These assays were done on location by the clinical centres recruiting the patients, using fresh plasma and cells.

### Serological testing

The South African cohorts underwent testing for Hepatitis B surface antigen (HBsAg) retrospectively from frozen sera using the Biokit enzyme immune assay (Barcelona, Spain). For Botswana, HBsAg results were performed in Gaborone, using the Murex HBsAg v3 (DiaSorin) assay.

HBsAg-positive samples with sufficient volume underwent testing for HBeAg by the chemilumiscence immune assays Architect (Abbott Diagnostics, Maidenhead, UK) (Durban cohorts) and ADVIA Centaur CP (Siemens, Camberley, UK) (Gaborone and Kimberley cohorts). We also investigated for potential HDV coinfection by screening HBsAg-positive samples for total HDV antibody when sufficient sample volume was available, using the DIA.PRO HDV Ab enzyme immune assay (Milan, Italy).

### HBV DNA load and sequencing

HBsAg-positive/HBeAg-negative samples for which sufficient sample was available underwent HBV DNA quantification by real-time PCR as previously described [22] (lower limit of quantification 50 IU/mL). Samples with HBV DNA >500 IU/ml underwent population (Sanger) sequencing of the HBV polymerase gene (amino acids 1–344) to determine genotype/sub-genotype and presence of drug-resistance associated mutations (RAMs), as previously described [22, 23]. HBV genotyping was initially done using the online automated subtyping tool Oxford HBV BioAfrica (http://www.bioafrica.net/rega-genotype/html/indexhbv.html) [24, 25]. To provide confirmation of these results and sub-genotyping, we undertook further phylogenetic analysis using a panel of reference sequences with maximum likelihood (ML) as implemented in PhyML 3.0 (1,000 bootstrap replicates) [26, 27].

### IFN-gamma ELISpot Assays

We undertook ELISpot assays using samples from the Durban HIV-infected cohort with sufficient available cells (n = 325, of whom 35 were positive for HBsAg (10.7%), and 10 were positive for HBeAg (28.6% of HBsAg-positives)). *Ex vivo* CD8+ T cell responses to HIV were quantified by testing peripheral blood mononuclear cells (PBMCs) against a panel of 410 overlapping HIV-1 peptides spanning the entire HIV-1 proteome, as previously described [19].

### Statistical analysis

Data were analysed using GraphPad Prism v.6.0f. Fisher’s Exact Test was used to compute significance for categorical variables in 2x2 contingency tables. HIV-1 RNA viral loads and CD4+ T cell counts in HBsAg-positive vs. HBsAg-negative subjects were compared using a Mann Whitney U test. HBsAg seroprevalence was quoted with 95% confidence intervals (CI) calculated by the adjusted Wald test (http://www.measuringu.com/wald.htm).

## Results

### HIV status

This study enrolled a total of 1,022 ART-naïve HIV-positive women from South Africa and Botswana. HIV subtype was not determined, but based on the origin of the patients is likely to have been C-clade in the majority [28]. In addition, we recruited 72 HIV-negative adults from Durban. Raw data for the cohort are available as supplementary data (S1 Table).

There were no significant differences in CD4+ T cell count between different sub-cohorts (S1 Fig; Table 1). HIV-1 RNA viral loads were higher in the South African cohorts (median RNA VL 30,800 copies/ml and 33,947 copies/ml in Durban and Kimberley, respectively) than in Botswana (median RNA VL 16,400 copies/ml; S1 Fig; Table 1).

### HBsAg prevalence

The overall prevalence of HBsAg in the combined cohort was 72/1022 (7.0%; 95% CI 5.6–8.8%; Table 1). Prevalence varied geographically: the lowest prevalence was in Botswana, where 17/443 (3.8%, 95% CI 2.3–5.9%) individuals tested positive for HBsAg, in contrast to South Africa (Durban and Kimberley cohorts), where 49/507 (9.7%, 95% CI 6.7–11.5%) were HBsAg positive.

The prevalence of HBsAg was not significantly different between HIV-positive vs. HIV-negative sub-groups (66/950 (6.9%) vs. 6/72 (8.3%); p = 0.6, Fisher’s Exact Test). Restricting this comparison to Durban (from where all our HIV-negative subjects were recruited), the prevalence of HBsAg was 40/426 (9.4%) versus 6/72 (8.3%) in HIV-positive and HIV-negative individuals respectively (p = 1.0).

### Virological characteristics of HBV infection in HIV-positive adults

To characterize the HBV replicative status of HIV/HBV coinfected patients, we first screened HBsAg-positive individuals for HBeAg. Overall, 16/60 (26.7%) HIV/HBV coinfected individuals were HBeAg positive (Table 1). Among 30 HBsAg-positive/HBeAg-negative individuals from South Africa and Botswana the HBV DNA load was median 1.8 log_10_ IU/ml (IQR 1.6–3.7 log_10_ IU/ml). Of these, 9/30 (30%) had HBV DNA >2,000 IU/ml. HDV total antibody was not detected in any of the 60 HBsAg-positive individuals tested (Table 1).

We sequenced the HBV polymerase gene from 16 individuals with detectable HBV DNA. Of these, 15/16 did not contain HBV drug RAMs, but one specimen harboured the M204I nucleos(t)ide analogue RAM. HBV genotypes were A for the South Africa cohort (9/14 (64%) sub-genotype A1 and 5/14 (36%) A2) and D for the Botswana cohort (2/2 (100%) sub-genotype D3).

### Impact of HBV co-infection on HIV-1 RNA load, CD4+ T-cell count and HIV-specific CD8+ T-cell responses

**Fig 1:**
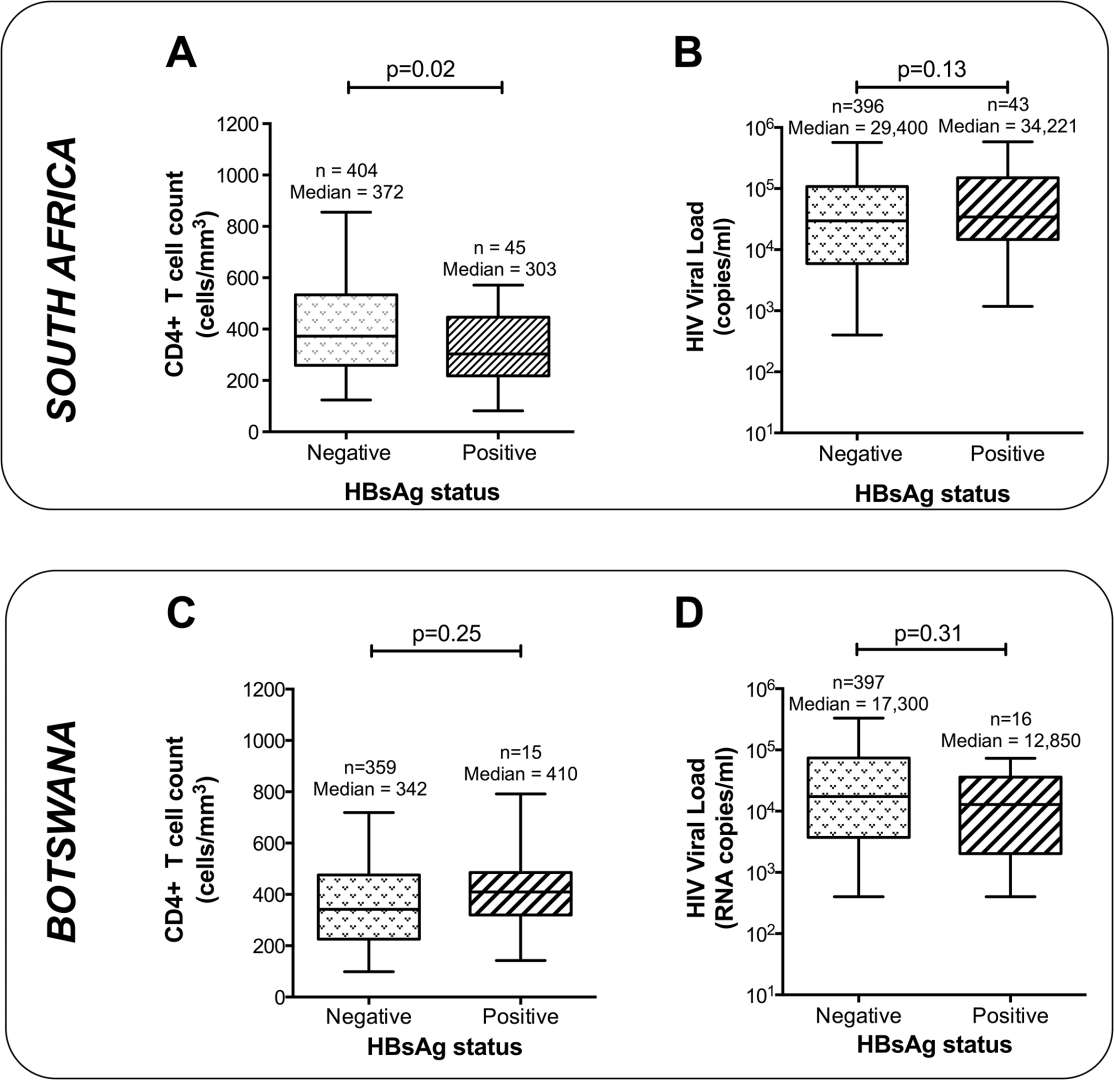
Relationship between HBV status and markers of HIV disease in HIV-positive women from South Africa and Botswana. Panels (A) and (B): South Africa (Durban + Kimberley cohorts pooled); Panels (C) and (D): Botswana (Gaborone). Left-hand column (panels (A) and (C)) shows CD4+ T cell counts; right-hand column (panels (B) and (D)) shows HIV-1 RNA viral load. In each case, box represents median and 25/75^th^ centiles, whiskers 5-95^th^ centiles. P values by Mann Whitney U test.

Within our combined HIV-positive South African cohort (Durban and Kimberley), HBsAg-positive individuals had statistically lower CD4+ T cell counts than HBsAg-negative individuals (303 vs. 372 cells/mm^3^, respectively; p = 0.02, Mann-Whitney test, Fig 1A). The median HIV-1 RNA load was slightly higher in HBsAg-positive individuals than in HBsAg-negative individuals in South Africa although this did not reach statistical significance (34,221 RNA copies/ml vs. 29,400 copies/ml; p = 0.13, Mann Whitney test; Fig 1B). No significant association was observed between HBV status and CD4+ T cell count or HIV-1 RNA load in Botswana (Fig 1C and 1D).

As CD8+ T cell responses are well recognized to be an important mediator of HIV disease control [19, 29, 30], we sought evidence of any impact of HBV coinfection on *ex vivo* CD8+ T cell responses to HIV using IFN-gamma ELISpot assays for 325 subjects from within the Durban cohort. There was no significant difference in the breadth, magnitude, or protein-specificity of IFN-gamma responses to HIV in the presence or absence of HBsAg or HBeAg (S2 Fig).

## Discussion

The virological expression of HBV infection varies substantially among HIV-positive individuals in sub-Saharan Africa, and its determinants remain poorly characterized [22]. This study determined the prevalence and virological expression of HBV coinfection in a composite cohort of ART-naïve HIV-positive adult women from South Africa and Botswana. Comparing our HBsAg prevalence data to other published literature is difficult, as data are relatively sparse and epidemiology varies by setting. However, broadly speaking, our current results are comparable to those assimilated from the review we have recently undertaken of this region [11]; the latter incorporated data on 11,346 HIV-positive individuals from southern Africa, of whom 687 (6.1%) were reported to have chronic HBV (median 6.0% across all studies). This study suggests that HDV is unlikely to be a major contributory factor to chronic liver disease in the specific settings we have studied, but further detailed work is needed to establish HDV hotspots and risk factors.

Interestingly, although CD4+ T cell counts were comparable between our geographically distinct sub-cohorts, plasma HIV-1 RNA levels were significantly lower in Botswana than South Africa, likely as a result of multiple influences that include the maturity of the epidemic and fitness of the viral infecting strain [17].

Our data point to a potential association between HBsAg-positivity and lower CD4+ T cell counts (Fig 1A), but this is a weak effect that does not appear consistent across locations; this is in keeping with uncertainty in the published literature to date [11]. Where this effect does operate, the direction of causality is uncertain; it is possible that chronic HBV infection may contribute to the decline of CD4+ T cell populations that are already diminished by HIV, but alternatively the effect could be explained by an increased risk of acquisition or reactivation of chronic HBV infection in HIV-positive individuals with low CD4+ T cell counts. Without individual level data, (e.g. duration of HIV infection), we are unable to draw any certain conclusions about the factors underpinning differences in CD4+ T cell counts between cohorts or by HBV status.

There are several caveats and limitations to our analysis. Due to the cross-sectional nature of the study, we were unable to verify the persistence of HBsAg over time; but for the purposes of this study we made the assumption that HBsAg-positivity represented chronic infection. It should be noted that we enrolled women attending antenatal clinics, and that pregnancy may modulate the nature of the immune response to viral infection; for example, it is recognized that HBV viral loads are typically higher in the second half of pregnancy. Although HIV-positive patients were all recorded as ‘ART-naïve’ at the time of enrollment, we cannot exclude the possibility that a minority of individuals may have accessed short courses of treatment prior to the study. Thus the detection of the M204I substitution may reflect either transmission of a RAM, or prior lamivudine exposure that was unreported by the patient.

It is evident that HIV/HBV coinfection patterns differ substantially by region [11], and an attempt to combine analysis of individuals from disparate settings could be misleading. Detailed demographic data were not recorded for these cohorts at the time of recruitment, so we are not able to draw conclusions regarding the specific relationship of our findings to different communities, regions and countries, or to age or socio-demographic characteristics.

To make the analysis as broad and far-reaching as possible, we avoided limiting ourselves to a single geographic cohort. In the interests of optimizing numbers, we have pursued the multi-centre approach, but to emphasise differences by region we have presented epidemiology data as separate results for individual sub-cohorts by region (Table 1; Fig 1; S1 Fig). Although we made every effort to optimize numbers, analysis of even greater numbers of subjects would be beneficial to form more robust conclusions.

Another influence on the epidemiology of this co-epidemic is the roll-out of the prophylactic HBV vaccination that has been variably introduced across sub-Saharan Africa in the past two decades; HBV prevalence will doubtless continue to change in accordance with the success of this vaccine campaign [31]. A recent review from South Africa points out that although vaccine coverage among children has increased since the mid 1990’s, there has been no ‘catch-up’ campaign, and vulnerable individuals remain unimmunized [32]. Other countries in the region have been slower to introduce routine vaccination [33, 34], and the coverage of vaccine campaigns is uncertain. Furthermore, because we did not test for serological markers of immunity (HBsAb or HBcAb) it is uncertain to what extent the HBsAg-negative individuals here may have acquired vaccine-mediated or natural immunity.

The difficulty of assimilating robust and representative data on the characteristics of HIV/HBV co-infection in this region is highlighted by this study; despite assimilating >1000 individuals over a ten year period, we identified only 72 with HBsAg-positive status. Furthermore, our dataset is incomplete, as small volumes within some of the samples meant that we were able to test only a subset of the whole cohort for HBeAg and HDV. There is no single systematic bias that we can identify that is likely to be clearly associated with this issue, but we are unable to categorically state the influence of missing data. We were also limited by availability of cells for *ex vivo* T cell studies: it remains possible that HBV-specific CD8+ T cell responses are abrogated by the presence of HIV, but we unfortunately did not have sufficient cells to investigate this possibility.

Despite these caveats and limitations, we believe this dataset to be of utility and importance in providing insight into the extent and characteristics of the HBV/HIV co-epidemic in certain southern African populations. Ongoing efforts are needed to secure prompt diagnosis and appropriate monitoring and treatment for coinfected individuals, in order to avert an emerging crisis of chronic liver disease.

## Supporting Information

S1 Fig Laboratory characteristics of HIV infection in adult women from three different HIV-1 infected cohorts. (A) HIV-1 RNA viral load and (B) CD4+ T cell count. Boxes show 25–75% centiles, whiskers show 5–95% CI. There was significant variation in HIV-1 RNA viral load between cohorts (p = 0.0002, Kruskal-Wallis test), but not in CD4+ T cell counts (ns = not significant).
(TIFF)

S2 Fig Relationship between HBsAg and HBeAg status and IFN-gamma ELISpot responses to HIV proteins in a cohort of 325 HIV-infected adult women from Durban, South Africa. (A): Total number of ELISpot responses across the entire HIV proteome according to HBsAg status; (B): Total number of HIV proteins targeted by ELISpot responses according to HBsAg status; (C): Number of Gag-specific ELISpot responses according to HBsAg status; (D): Magnitude of immunodominant ELISpot response according to HBsAg status; (E): Total number of ELISpot responses across the entire HIV proteome according to HBeAg status; (F): Total number of HIV proteins targeted by ELISpot responses according to HBeAg status; (G): Number of Gag-specific ELISpot responses according to HBeAg status; (H): Magnitude of immunodominant ELISpot response according to HBeAg status. Error bars show 95% CI. P-values by Mann Whitney U test.
(TIFF)

S1 Table Spreadsheet containing raw data for 1,022 southern African women (tab 1), and for the subset of 72 women testing HBsAg-positive (tab 2). Tab 1 contains cohort location, HIV status, HBsAg status, HIV-1 RNA viral load (copies / ml plasma) and CD4+ T cell count (cells/mm^3^). Tab 2 contains HBeAg status, HDV antibody status, HBV DNA viral load (IU/ml) and HBV genotype.
(XLSX)
