# Supplementary figures and images for: Prevalence and Characteristics of Hepatitis B Virus (HBV) Coinfection among HIV-Positive Women in South Africa and Botswana
Source: PLoS One. 2015 Jul 28;10(7):e0134037. doi: 10.1371/journal.pone.0134037 (PMC4517770; doi:10.1371/journal.pone.0134037)

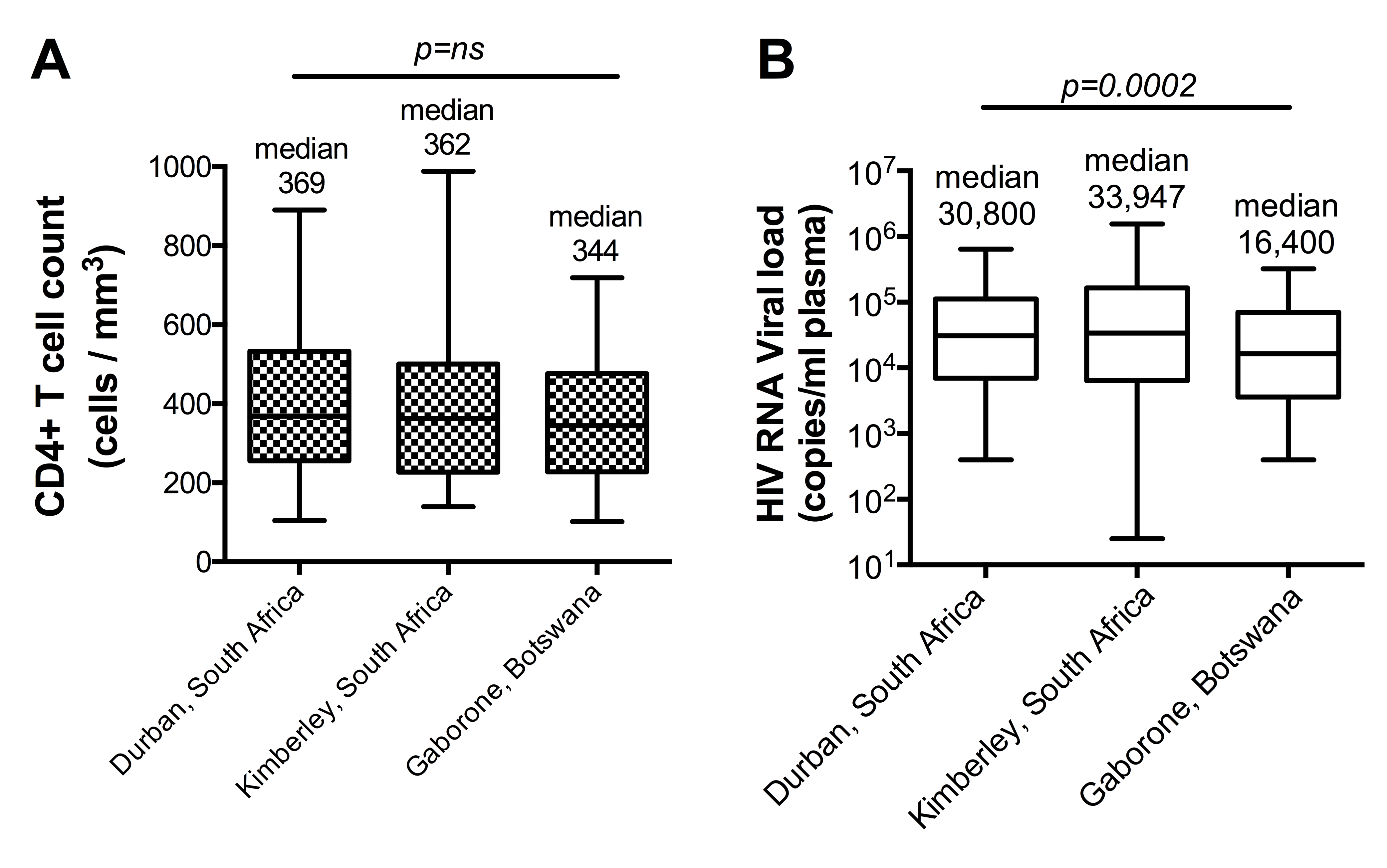

Supplement: S1 Fig — (A) HIV-1 RNA viral load and (B) CD4+ T cell count. Boxes show 25–75% centiles, whiskers show 5–95% CI. There was significant variation in HIV-1 RNA viral load between cohorts (p = 0.0002, Kruskal-Wallis test), but not in CD4+ T cell counts (ns = not significant). (TIFF) [file pone.0134037.s001.tiff]

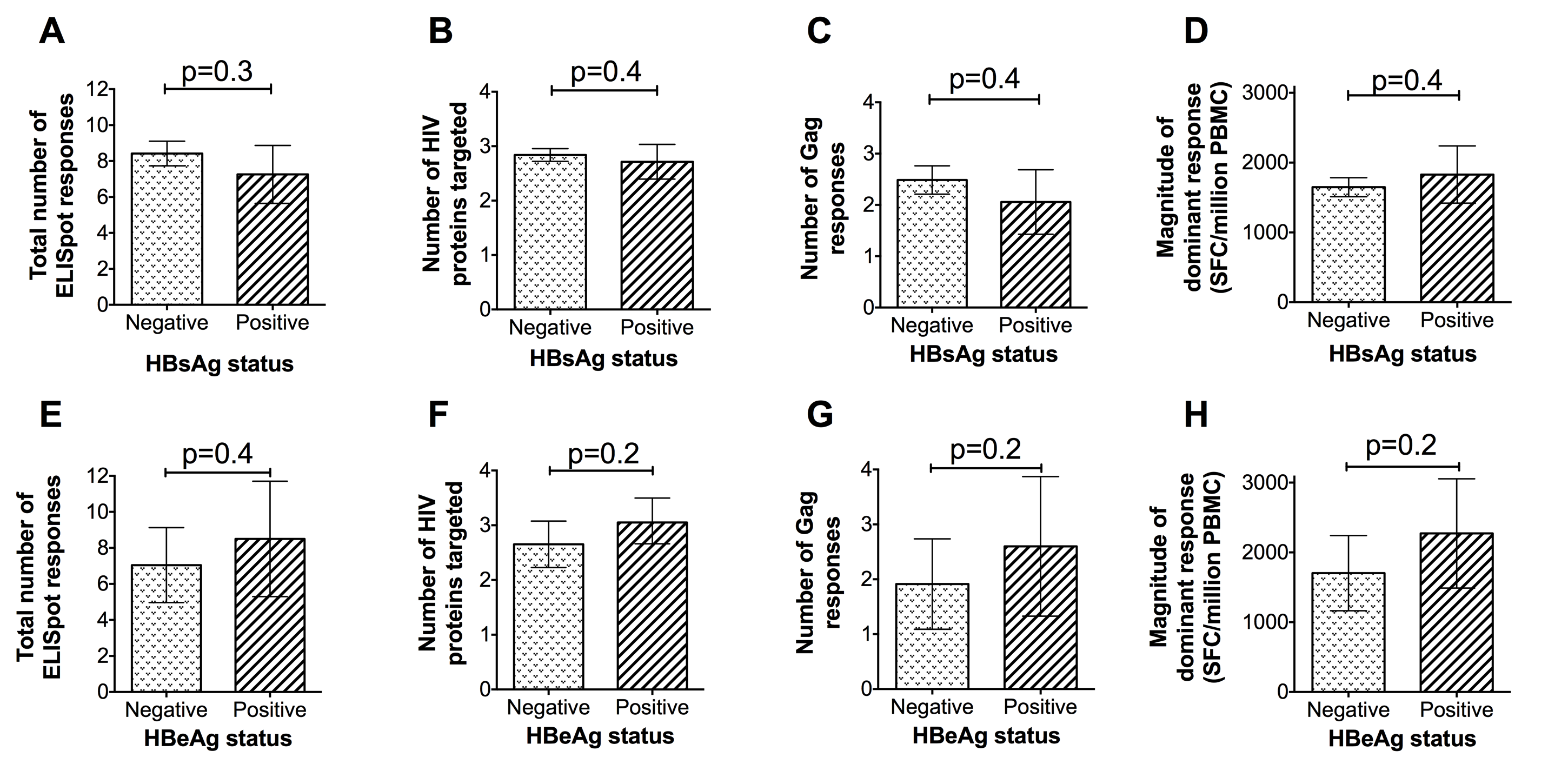

Supplement: S2 Fig — (A): Total number of ELISpot responses across the entire HIV proteome according to HBsAg status; (B): Total number of HIV proteins targeted by ELISpot responses according to HBsAg status; (C): Number of Gag-specific ELISpot responses according to HBsAg status; (D): Magnitude of immunodominant ELISpot response according to HBsAg status; (E): Total number of ELISpot responses across the entire HIV proteome according to HBeAg status; (F): Total number of HIV proteins targeted by ELISpot responses according to HBeAg status; (G): Number of Gag-specific ELISpot responses according to HBeAg status; (H): Magnitude of immunodominant ELISpot response according to HBeAg status. Error bars show 95% CI. P-values by Mann Whitney U test. (TIFF) [file pone.0134037.s002.tiff]
